# Muscle phenotype is related to motor unit behavior of the vastus lateralis during maximal isometric contractions

**DOI:** 10.14814/phy2.13636

**Published:** 2018-03-11

**Authors:** Ryan J. Colquhoun, Mitchel A. Magrini, Cody T. Haun, Tyler W. D. Muddle, Patrick M. Tomko, Micheal J. Luera, Cameron S. Mackey, Christopher G. Vann, Jeffrey S. Martin, Kaelin C. Young, Jason M. DeFreitas, Michael D. Roberts, Nathaniel D. M. Jenkins

**Affiliations:** ^1^ Applied Neuromuscular Physiology Laboratory Oklahoma State University Stillwater Oklahoma; ^2^ Molecular and Applied Sciences Laboratory Auburn University Auburn Alabama; ^3^ Department of Cell Biology and Physiology Edward Via College of Osteopathic Medicine ‐ Auburn Campus Auburn Alabama

**Keywords:** motor unit firing rates, myosin heavy chain, sEMG decomposition

## Abstract

Previous investigations have reported a relationship between skeletal muscle phenotype and motor unit (MU) firing parameters during submaximal contractions. The purpose of the current investigation, however, was to examine the relationships between motor unit firing behavior during a maximal voluntary contraction, Myosin Heavy Chain (MHC) isoform content, and various molecular neuromuscular targets of the vastus lateralis (VL) muscle in resistance‐trained men. Ten resistance‐trained males completed a trapezoidal ramp contraction up to 100% of their maximal voluntary isometric strength (MVIC). Surface electromyography was recorded from the VL using a multichannel electrode array and decomposed to examine the firing characteristics of individual MUs. A skeletal muscle biopsy of the VL was also collected from each subject. Regression analyses were performed to identify relationships between type II fiber area and the slopes and/or intercepts of the mean firing rate (FR_MEAN_) versus recruitment threshold (RT), max firing rate (FR_MAX_) versus RT, and RT versus MU action potential amplitude (MUAP_PP_) relationships. There were significant inverse relationships between type II fiber area and the *y*‐intercept of the FR versus RT relationship (*P* < 0.05). Additionally, strong relationships (*r* > 0.5) were found between type II fiber area and FR_MEAN_ versus RT slope and RT versus MUAP_PP_ slope and intercept. These data further support the hypothesis that skeletal muscle phenotype is related to MU behavior during isometric contraction. However, our data, in concert with previous investigations, may suggest that these relationships are influenced by the intensity of the contraction.

## Introduction

Skeletal muscle force production is accomplished via the progressive recruitment of motor units (MU) and modulation of their firing rates (i.e., rate coding). While it is well established that MUs are recruited in an orderly fashion based on soma size (i.e., the size principle (Henneman 1957)), there has been some controversy surrounding the relationship between MU recruitment thresholds and MU firing rate behavior with increasing force production (Moritz et al. 2005; Oya et al. 2009). However, evidence suggests that an inverse relationship exists between a MU's recruitment threshold and its firing rate during voluntary contractions. This pattern has been titled the onion‐skin motor unit control scheme (De Luca and Erim 1994; De Luca and Hostage 2010) and is particularly appealing for human motor control in that it theoretically favors force steadiness, control, and sustained force production, as opposed to maximal force production (Hu et al. 2014b; De Luca and Contessa 2015). It has also been established that the central nervous system (CNS) does not control the behavior of individual MUs, but rather modulates the entire motor neuron pool with a common synaptic input (i.e., common drive) from the CNS (De Luca and Erim 1994; Farina et al. 2014). Furthermore, it has been suggested that during voluntary contractions, the excitation input to the MU pool is adjusted to maintain force production in response to increasing (i.e., potentiation) or decreasing (i.e., fatigue) MU twitch amplitudes (de Luca et al. 1996; Contessa et al. 2016; Trevino et al. 2016). Thus, the resulting firing behavior of the motor unit is determined, at least in part, by its intrinsic physical properties.

Although myoblast cell lineage plays an important role in muscle phenotype independent of external influences (Düsterhöft and Pette 1993; Gundersen 1998), it has also been established that external influences such as the electrical activity and/or chemical substances released from the motor neuron innervating skeletal muscle fibers likely have a strong influence on muscle phenotype as well. For example, when motor neurons that innervate a ‘slow’ muscle (i.e., soleus) are cross‐innervated to innervate a “fast” muscle in animal models, the contractile speed of the “fast” muscle (i.e., flexor digitorum longus) decreases (Buller et al. 1960), whereas the time characteristics (i.e., duration of after‐hyperpolarization, conduction velocity) of the motor neurons themselves do not change. Therefore, Buller et al. (1960) suggested that the cross‐innervated motor neurons change the contractile speed of the muscle such that the relationship between the frequency characteristics of the motor neuron and the muscle fibers are optimal. In a later study, Lomo et al. (1974) demonstrated that muscle contractile speed is modified based on the pattern of electrical activity, independent of a motor neuron or supposed neurotrophic substances. This was later supported by the work of Gardiner and Kernell (1990), who suggested that the contractile speeds of muscle fibers corresponded with the properties of the innervating motor neuron in both cat and rat models. Consequently, several authors have hypothesized a direct relationship between fiber‐type composition (i.e., Myosin Heavy Chain (MHC) content) and MU firing behavior (Orizio and Veicsteinas 1992; Beck et al. 2007; De Luca and Contessa 2012; Trevino and Herda 2016).

In support of this hypothesis, both De Luca et al. (1982) and Herda et al. (2015) have reported differences in the MU control properties between individuals of different training backgrounds. For instance, De Luca et al. (1982) reported significant differences in the firing rates of the deltoid and first dorsal interosseous in Olympic‐caliber weightlifters and swimmers, concert pianists, and healthy control subjects. Examining the regression coefficients of the relationships between the recruitment thresholds and firing rate of the detected motor units provides significant insight into the overall control scheme utilized by a muscle to regulate force production. Using this method, Herda et al. (2015) reported significantly higher mean firing rates (FR_MEAN_) across the recruitment threshold (RT) spectrum (i.e., greater *y*‐intercepts) during isometric contractions at a targeted force level of 40% and 70% maximal voluntary isometric contraction strength (MVIC) in endurance‐trained versus chronically resistance‐trained individuals. Thus, it appears that training status, and presumably skeletal muscle phenotype, influence MU firing behavior. Indeed, Herda et al. (2015) hypothesized that the differences in firing rates between endurance and resistance‐trained individuals were due to potential differences in fiber‐type distribution between groups, as it would be assumed the endurance‐trained individuals exhibited a higher Type I MHC content than their resistance‐trained counterparts.

In a follow‐up investigation, Trevino et al. (2016) examined the influence of Type I MHC on MU firing behavior of the vastus lateralis in vivo during a submaximal isometric contraction at 40% MVIC. The authors reported that regardless of skeletal muscle phenotype, all subjects displayed a motor unit control scheme consistent with the onion skin theorem. However, the authors also reported less negative slopes for those subjects with higher Type I MHC content. These results suggested that subjects with higher Type I MHC content exhibit a shallower slope in the FR_MEAN_ versus RT relationship, which in turn suggests that subjects with higher Type I MHC content exhibit a smaller difference between the firing rates for lower versus higher threshold MUs at a submaximal target force (40% MVIC) than those with higher Type II MHC content (Trevino et al. 2016). Therefore, Trevino et al. (2016) provided data that suggested a definitive relationship between MHC content and MU firing behavior during low‐intensity contractions in vivo. An important but often overlooked characteristic of MU firing behavior is the *y*‐intercept of the FR_MEAN_ versus RT relationship, which has been suggested as an indicator of the maximal sustainable firing rate of the lowest threshold MUs in the motor neuron pool (Trevino et al. 2016). Interestingly, Trevino et al. (2016) did not observe a relationship between muscle fiber type and the *y*‐intercept of the FR_MEAN_ versus RT relationship, although Herda et al. (2015) previously observed differences in the *y*‐intercepts of the FR_MEAN_ versus RT relationship in endurance versus resistance‐trained individuals. Therefore, Trevino et al. (2016) hypothesized that factors such as training status may also play a role in determining motor unit behavior. Thus, the relationship between MHC content and MU firing during high‐intensity contractions remains unknown and it remains unclear how or if fiber‐type composition is related to motor unit behavior in a sample of subjects with a similar training background.

The purpose of the current investigation, therefore, was to examine the relationships between motor unit firing behavior during a maximal voluntary contraction, MHC isoform content, and various molecular neuromuscular targets of the vastus lateralis muscle in resistance‐trained men. On the basis of the results of Herda et al. (2015) and Trevino et al. (2016) we hypothesized that subjects possessing greater Type I MHC content would exhibit a greater *y*‐intercept and a less negative mean firing rate versus recruitment threshold slope than those with lower Type I MHC content.

## Methods

### Ethical approval

Participants were informed of all experimental procedures and completed a written informed consent document before participating in the present investigation. The study's procedures were approved by Auburn University's Institutional Review Board (Protocol #16‐333 MR 1609) in accordance with the Declaration of Helsinki.

### Participants

Ten healthy, resistance‐trained males (mean ± SD, age = 22 ± 2 years; height = 180.6 ± 5.1 cm; weight = 87.1 ± 10.9 kg) volunteered to participate in this investigation. Participants were defined as resistance‐trained if they had been actively participating (≥3 days·week^−1^) in resistance training for a minimum of six consecutive months. Participants reported no history of neurological illness or musculoskeletal injury of the lower extremities prior to participating in this investigation.

### Motor unit recordings, processing, and analysis

**Figure 1:**
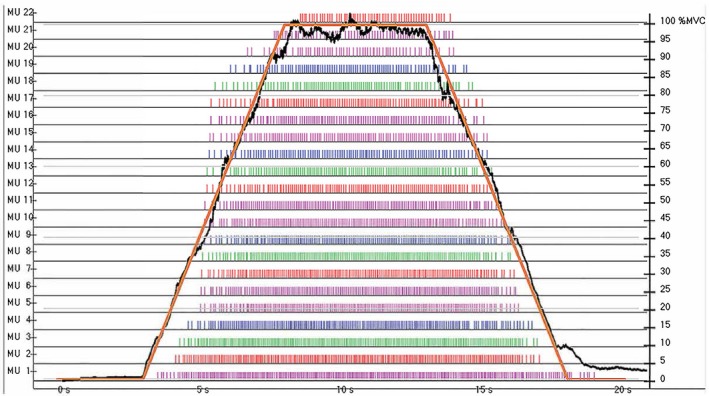
An example of a maximal voluntary isometric contraction (MVIC) ramp contraction (MVIC_RAMP_). The force trajectory increased linearly at 20% of max force (*F*_MAX_)·sec^−1^, plateaued and was held at *F*_MAX_ for 6 sec, and then decreased linearly at 20% *F*_MAX_·sec^−1^ back to baseline, with the subject's actual force output overlaid.

All maximal voluntary isometric contraction (MVIC) testing took place with the participants seated in a custom‐built chair so that their hip and knee joints were positioned at approximately 90° angles. A padded ankle strap (Spri Products, Inc., Libertyville, IL, USA) was wrapped approximately 3 cm above the lateral malleolus of the participant's right ankle and was attached in series to a force transducer (Model SSM‐AJ‐500; Interface, Scottsdale, AZ, USA) which was anchored to the custom built chair. Following a thorough explanation of the testing procedures, participants warmed‐up by completing several submaximal contractions of the knee extensor musculature. Each participant then completed 2 MVIC attempts, with 1 min between attempts to avoid any undue fatigue. If the second MVIC attempt was higher than the first, a third attempt was given. However, no participant further increased force output during their third MVIC and thus, no one completed more than three MVIC attempts during testing. For all MVIC attempts, participants were instructed to “kick out as hard as possible” for approximately 5 sec. The highest force (*F*_MAX_), calculated as the highest average force value that occurred during a 1000 msec epoch, achieved during these attempts was recorded and utilized as the target force for the force trajectory in subsequent testing. Following the completion of MVIC testing, participants completed an MVIC ramp contraction (MVIC_RAMP_) by tracing a trapezoidal‐shaped force trajectory. Specifically, the force trajectory increased linearly at 20% *F*_MAX_·sec^−1^, plateaued and was held at *F*_MAX_ for 6 sec, and then decreased linearly at 20% *F*_MAX_·sec^−1^ back to baseline. An example MVIC_RAMP_ and corresponding MU firing behavior is presented in Figure 1 below. Upon completion of the MVIC_RAMP_, the contraction was visually inspected by the same investigator for compliance. If the contraction was deemed unsuccessful (i.e., the participant did not accurately follow the force trajectory), the participant was given adequate rest before completing another MVIC_RAMP_. No participant required more than 2 MVIC_RAMP_ contractions during testing.

During each contraction, surface electromyography (sEMG) signals were collected from the participant's vastus lateralis using a specialized five‐pin sensor and a 16‐channel Bagnioli acquisition unit (Delsys Inc., Natick, MA, USA). Prior to the sensor placement the skin was prepared by carefully shaving, abrading, and cleansing of the area with alcohol. The five‐pin sensor was then secured to the vastus lateralis with hypoallergenic tape in accordance with the recommendations set forth by Zaheer et al. (2012). A reference electrode (Dermatrode; American Imex, Irvine, CA, USA) was placed over the spinous process of the C7 vertebrae and secured with hypoallergenic tape. The four channels of raw sEMG signal recorded during each contraction were stored on a personal computer and decomposed offline using the Precision Decomposition III Algorithm described by De Luca et al. (2006) and improved upon by Nawab et al. (2010). Following the decomposition process, only MU's demonstrating at least 90.0% accuracy (as determined by the Decompose‐Synthesize‐Decompose‐Compare Test) were retained and used in subsequent analysis. Additionally, only contractions demonstrating a recruitment threshold (RT) range of at least 20% MVIC were included in the final MU analysis.

All MU firing rate curves were smoothed prior to calculation by low‐pass filtering each motor unit's impulse train with a 2‐sec Hanning window. Custom‐written LabVIEW programs (LabVIEW 2015; National Instruments, Austin, TX, USA) were then used to analyze the MU's that met the inclusion criteria described previously. The MU properties calculated by the LabVIEW programs from each contraction included: (1) RT, which was defined as the relative force (% MVIC) at which the MU first discharged; (2) FR_MEAN_, which was calculated of as the average firing rate (pulses·sec^−1^ (pps)) during the plateau in each individual MU's firing curve; (3) max firing rate (FR_MAX_), calculated as the maximum rate of discharge (pps) of the firing rate curve for each individual MU during the contraction; and (4) MUAP_PP_, defined as the average peak‐to‐peak amplitude (mV) of the waveforms across the four sEMG channels.

### Muscle biopsy and fiber typing procedure

**Figure 2:**
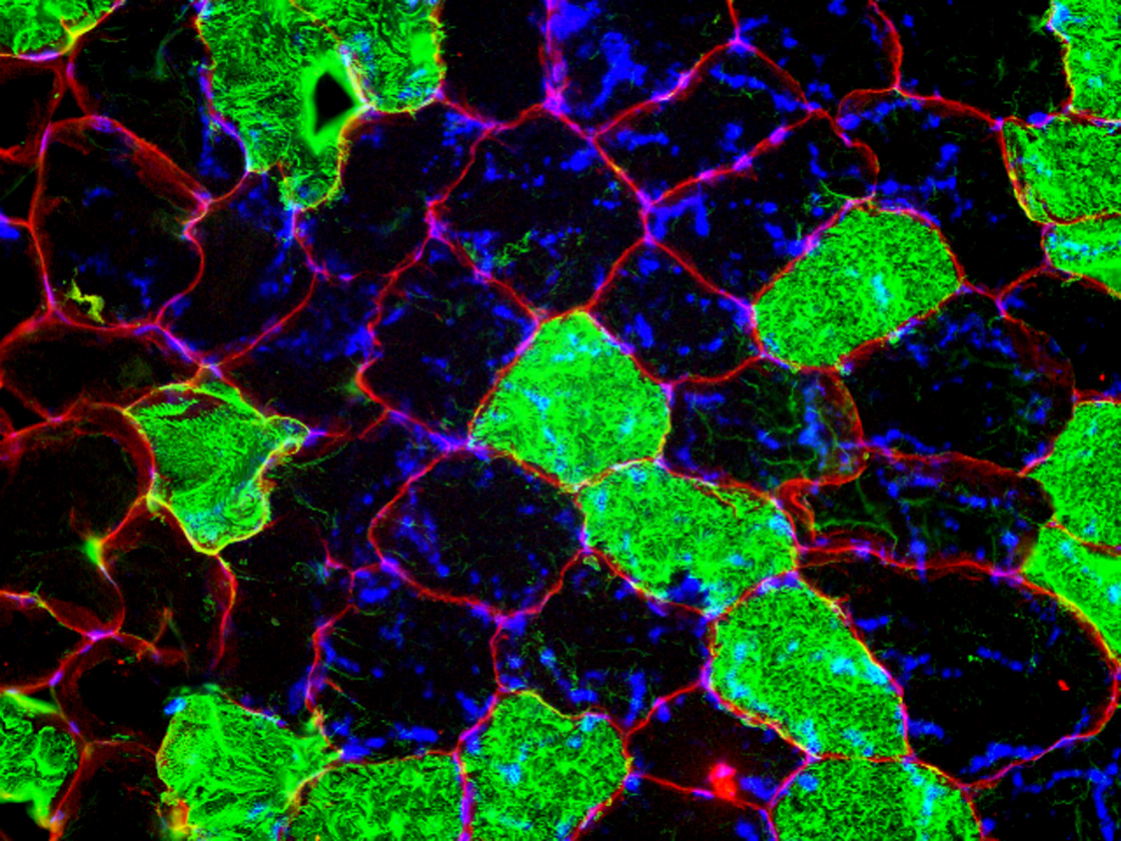
An imaged of a stained samples of muscle from the vastus lateralis. The outlined red represents dystrophin and blue represents nuclei, whereas the black and green stains represent type I and type II myosin heavy chain content (MHC), respectively.

Following MU recordings, participants laid in a supine position on a treatment table, whereby a percutaneous skeletal muscle biopsy was obtained from the right vastus lateralis midway between the patella and iliac crest using a 5‐gauge needle with suction and sterile laboratory procedures as described previously (Kephart et al. 2015). Briefly, 1.5 mL of 1% lidocaine was injected subcutaneously above the skeletal muscle fascia prior to making a small pilot incision for needle intrusion. The biopsy needle was then inserted at a depth just beyond the fascia, and approximately 100–150 mg of skeletal muscle was removed using a double‐chop method and applied suction. Biopsy samples were homogenized via a micropestle in 500 *μ*L of nondenaturing cell lysis buffer (Cell Signaling, Danvers, MA, USA) with protease and phosphatase inhibitors. Insoluble proteins were removed by centrifugation for 5 min at 500 g and a temperature of 4°C. Muscle homogenates were assayed for total protein content using a BCA Protein Assay Kit (Thermo Fisher Scientific, Waltham, MA, USA). Homogenates were subsequently prepared for western blotting by concentrating preparations at 1 *μ*g of protein (requisite volume calculated from BCA Assay) per 1 *μ*L of 4× Laemmli buffer so that equal concentrations of total protein were compared between subjects. 25 *μ*L of these preparations were loaded onto 4–15% SDS‐polyacrylamide gels (BioRad, Hercules, CA, USA) and subjected to electrophoresis (180 V for 60 min) using 1× SDS PAGE running buffer (Ameresco, Solon, OH, USA). Thereafter, proteins were transferred to polyvinylidene difluoride membranes (BioRad), Ponceau stained to correct for any differences in loading volumes between wells, and imaged using a gel documentation system (UVP, Upland, CA, USA). Membranes were then blocked for 1 h at room temperature with 5% nonfat milk powder. Membranes were incubated in 15 mL of TBST, 5% (in grams) bovine serum albumin powder (BSA), and the following primary antibodies at concentrations of 0.5 *μ*g antibody concentrations/mL of solution, separately: Na^+^–K^+^ ATPase *α*‐subunit (a5) (deposited to the DSHB by Fambrough, D.M. (DSHB Hybridoma Product a5)), Na^+^–K^+^ ATPase *β*‐subunit (24) (deposited to the DSHB by Fambrough, D.M. (DSHB Hybridoma Product 24)), and creatine kinase (CK‐1F7) (deposited to the DSHB by Morris, G.E. (DSHB Hybridoma Product CK‐1F7). Membranes were rocked overnight at 4°C. The next day, membranes were washed three times (5 min each wash) in TBST and incubated and rocked with horseradish peroxidase‐conjugated anti‐mouse IgG (1:2000, Cell Signaling, #7076) at room temperature for 1 h prior to membrane development. Membranes were then washed three times (5 min each wash) in TBST, development was performed using an enhanced chemiluminescent reagent (Luminata Forte HRP substrate; Millipore, Billerica, MA, USA), and band densitometry was performed using a gel documentation system and associated densitometry software (UVP). Band densities were normalized to Ponceau stains, and these values are presented as arbitrary density units. In order to fiber type each subject, images of the stained fibers (see Fig. 2 for example) were uploaded into Adobe Photoshop (Abode, Inc., San Jose, CA, USA). The same investigator individually counted and recorded the number of Type I and Type II fibers stained in each image. This process was then repeated for a second trial. If there was any disagreement between the first two trials, a third trial was conducted and the two trials closest in value were averaged and recorded as the fiber‐type distribution for that subject.

### Statistical analysis

Normality of the data was assessed using Shaprio–Wilk tests. Linear regressions were performed to analyze the following relationships for each individual subject: FR_MEAN_ (pps) versus RT (%MVIC), FR_MAX_ (pps) versus RT (%MVIC), and RT (%MVIC) versus MUAP_PP_ (*μ*V). Slopes and *y*‐intercepts were calculated for each linear regression model. Both significant and nonsignificant relationships were used in the final analysis. Subsequently, Pearson's product‐moment correlation coefficients were calculated to examine the relationships between the slopes and intercepts for the regression models of the above MU properties and Type II fiber area of the vastus lateralis (VL). Additional Pearson's product‐moment correlation coefficients were calculated to examine relationships among Type II fiber area of the VL, creatine kinase (CK) content, ATPase‐*α* content, ATPase‐*β* content, FR_MEAN_, FR_MAX_, and MVIC strength. Pearson's product‐moment correlations were characterized as small (*r* ≤ 0.10), moderate (0.10 > *r* < 0.50), or large (*r* ≥ 0.50) (Cohen 1992). Slope and *y*‐intercepts were calculated using Microsoft Excel^®^ 2015 (Microsoft, Inc., Redmond, WA). All statistical analyses were performed in SPSS Version 24 (IBM, Armonk, NY) with the level of significance set at *P* ≤ 0.05. Unless otherwise noted, data are presented as mean ± standard deviation (SD).

## Results

All data were normally distributed (*P* = 0.101–0.985). MUs were analyzed as a function of RT, as suggested by Trevino et al. (2016). The number of analyzed MUs, RT Range, MVIC, fiber‐type composition (relative area), the content of each neuromuscular target, as well as the slope and intercept of the FR_MEAN_ versus RT, FR_MAX_ versus RT, and RT versus MUAP_PP_ relationships for each individual subject are provided in Table 1.

**Table 1:** Individual subject data for maximal voluntary isometric contraction (MVIC) strength, motor unit (MU) yield, recruitment threshold (RT) range, fiber‐type area, and neuromuscular target (creatine kinase [CK], adenosine trisphosphatase [ATPase]) contents, as well as the slope, intercept, and *r* value for the MU behavior relationships assessed. The mean ± SD for each corresponding variable is listed below the body of the table

									FRMean versus RT			FRMax versus RT			RT versus MUAPpp		
Sub.	MVIC (N)	MU yield	RT range	Type I % Area	Type II % Area	CK	ATPase *α*	ATPase *β*	Slope	Intercept	Pearson	Slope	Intercept	Pearson	Slope	Intercept	Pearson
1	1198.5	23	28.6–87.9	22.41	77.59	2.81	9.94	1.57	−0.27	28.88	−0.84	−0.26	26.44	−0.82	0.13	15.43	0.91
2	969.1	22	15.2–59.2	51.18	48.82	2.40	13.86	1.57	−0.31	29.15	−0.89	−0.35	34.95	−0.88	0.16	14.58	0.83
3	1151.6	25	29.0–74.5	51.87	48.13	4.19	20.39	1.43	−0.37	34.30	−0.82	−0.41	40.19	−0.81	0.08	27.14	0.85
4	898.1	22	8.3–82.6	26.07	73.93	5.40	4.69	0.96	−0.18	22.10	−0.93	−0.20	28.05	−0.93	0.49	−3.95	0.71
5	894.7	26	1.4–56.0	48.98	51.02	6.42	9.63	1.75	−0.23	29.02	−0.94	−0.22	35.56	−0.92	0.24	−9.06	0.83
6	692.3	11	12.5–87.2	33.54	66.46	2.42	7.74	0.88	−0.06	15.28	−0.85	−0.08	23.98	−0.92	0.36	5.77	0.83
7	829.6	19	0.1–94.2	24.94	75.06	2.39	7.51	1.40	−0.11	19.88	−0.81	−0.15	27.16	−0.88	0.33	−4.68	0.64
8	1192.1	13	11.4–39.4	22.40	77.60	4.16	8.94	2.19	−0.27	21.21	−0.92	−0.37	28.37	−0.91	0.08	6.84	0.79
9	812.3	19	0.1–94.2	17.16	82.84	3.61	4.21	1.43	−0.15	22.13	−0.92	−0.14	27.56	−0.94	0.66	−35.50	0.92
10	945.0	18	9.1–44.7	43.80	56.20	3.20	15.06	1.65	−0.46	30.75	−0.95	−0.56	38.30	−0.96	0.15	5.88	0.83
Mean	958.3	19.8	11.6–72.0	34.2	65.80	3.70	10.20	1.48	−0.24	25.27	−0.89	−0.27	31.06	−0.90	0.27	2.25	0.81
SD	172.0	4.9	10.5–20.6	13.5	13.5	1.37	4.97	0.37	0.12	5.95	0.05	0.15	5.64	0.05	0.19	17.10	0.08

### FR_MEAN_ versus RT

All 10 subjects had a significant FR_MEAN_ versus RT relationship (*P* < 0.05; See Table 1). Additionally, the mean ± SD slope was −0.24 ± 0.12 pps·%MVIC^−1^, with a *y*‐intercept of 25.3 ± 5.9 pps.

**Figure 3:**
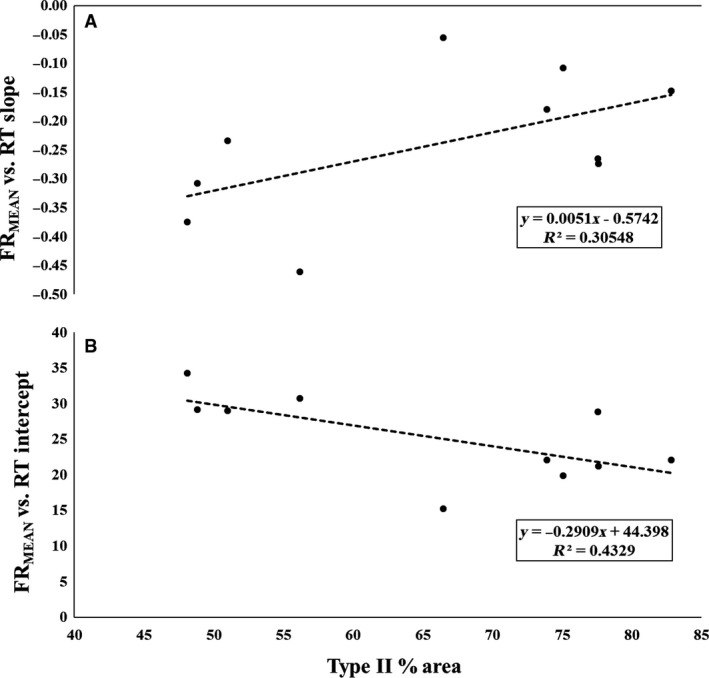
The plotted relationships between type II fiber area and the mean firing rate (FR_Mean_) versus (A) Recruitment Threshold (RT) slopes and (B) RT intercepts.

The results of the bivariate correlation table revealed a significant negative relationship between type II fiber area and FR_MEAN_ versus RT *y*‐intercept (*P* = 0.039; *r* = −0.658; Fig. 3B). Additionally, a strong positive relationship was found between type II fiber area and FR_MEAN_ versus RT slope (*P* = 0.101; *r* = 0.549; Fig. 3A). Our results also showed a strong negative relationship between average FR_MEAN_ and type II fiber area (*P* = 0.094; *r* = −0.558), as well as average FR_MAX_ and type II fiber area (*P* = 0.100; *r* = −0.549).

### FR_MAX_ versus RT

All 10 subjects had a significant FR_MAX_ versus RT relationship (*P* < 0.05; See Table 1). The mean ± SD slope was −0.27 ± 0.15 pps·RT^−1^ and the *y*‐intercept was 31.1 ± 5.6 pps.

**Figure 4:**
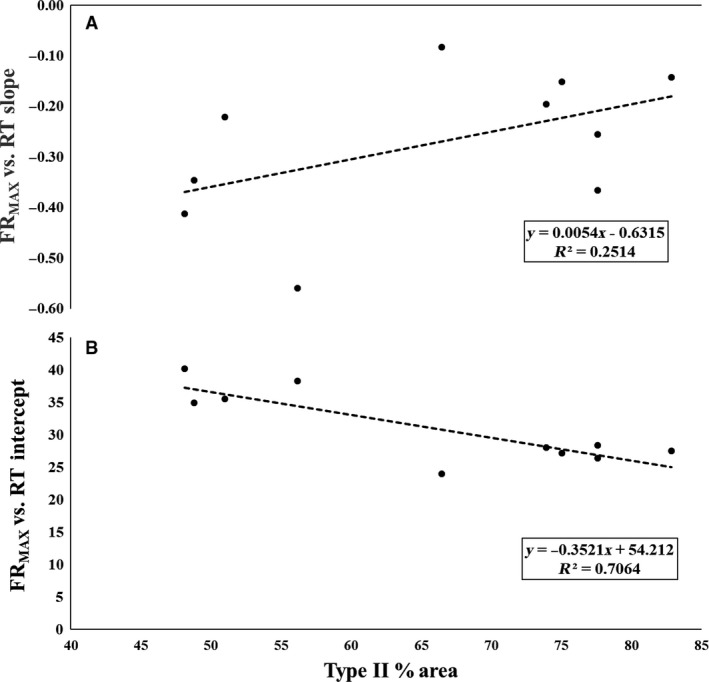
The plotted relationships between type II fiber area and the maximal firing rate (FR_MAX_) versus (A) Recruitment Threshold (RT) slopes and (B) RT intercepts.

There was a significant negative relationship between type II fiber area and FR_MAX_ versus RT *y*‐intercept (*P* = 0.002; *r* = −0.840; Fig. 4B), as well as, a moderate positive relationship between type II fiber area and FR_MAX_ versus RT slope (*P* = 0.145; *r* = 0.495; Figure 4A).

### RT versus MUAP_PP_

All 10 subjects displayed a significant RT versus MUAP_PP_ relationship (*P* < 0.05; See Table 1). The mean ± SD slope was 0.27 ± 0.19%MVIC·MUAP_PP_^−1^, with a *y*‐intercept of 2.25 ± 17.1%MVIC.

**Figure 5:**
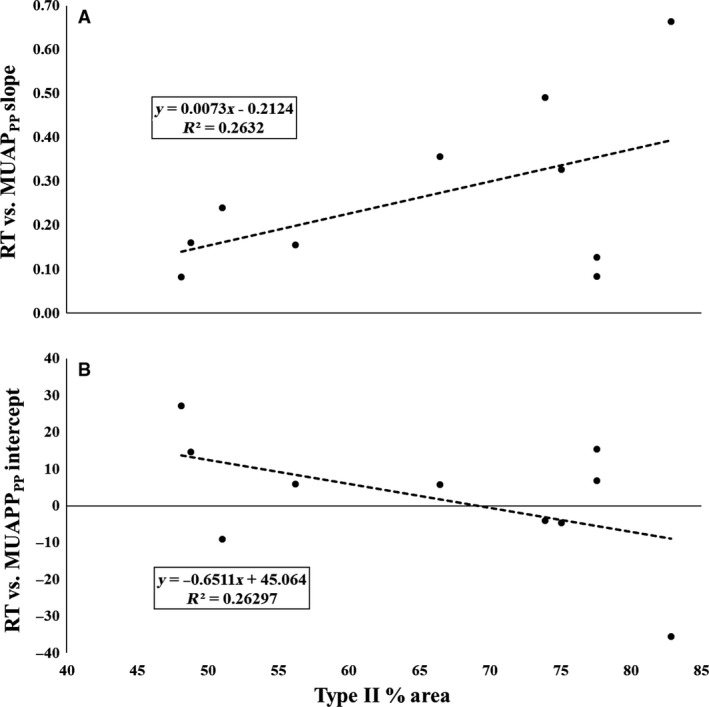
The plotted relationships between type II fiber area and the recruitment threshold (RT) versus (A) motor unit action potential amplitude (MUAP_PP_) slope and (B) MUAP_PP_ intercept relationships.

There was a strong, negative relationship between type II fiber area and RT versus MUAP_PP_ *y*‐intercept (*P* = 0.130; *r* = −0.513; Fig. 5B), as well as a strong positive relationship between type II fiber area and RT versus MUAP_PP_ slope (*P* = 0.128; *r* = 0.515; Fig. 5A).

### Neuromuscular targets

We observed a significant negative relationship between ATP‐*α* subunit content and type II fiber area (*P* = 0.007; *r* = −0.783). No significant relationships were observed between type II fiber area and either ATP‐*β* subunit (*P* = 0.854; *r* = −0.067) or creatine kinase content (*P* = 0.644; *r* = −0.167). Significant relationships were observed for ATP‐*α* subunit content and: FR_MEAN_ versus RT slope (*P* = 0.007; *r* = −0.785), FR_MEAN_ versus RT intercept (*P* = 0.007; *r* = 0.789), FR_MAX_ versus RT slope (*P* = 0.011; *r* = −0.758), FR_MAX_ versus RT intercept (*P* = 0.003; *r* = 0.832), RT versus MUAP_PP_ slope (*P* = 0.010; *r* = −0.764), and RT versus MUAP_PP_ intercept (*P* = 0.010; *r* = 0.763). Additionally, a significant correlation was found between MVIC strength and ATP‐*β* subunit (*P* = 0.049; *r* = 0.634).

## Discussion

The primary finding of the current investigation was the observed relationship between type II fiber area and MU firing behavior of the vastus lateralis during a maximal contraction. Specifically, our analyses revealed that all subjects demonstrated an inverse relationship between MU firing rate and threshold force, where earlier recruited MUs had greater firing rates than later recruited MUs. Thus, the onion skin control scheme was observed for all subjects. However, we also observed a significant, strong inverse relationship between type II fiber area and FR_MEAN_ versus RT *y*‐intercept, and a nonsignificant, but strong direct relationship between type II fiber area and FR_MEAN_ versus RT slope. Similarly, there was a significant strong inverse relationship between type II fiber area and FR_MAX_ versus RT *y*‐intercept, and a nonsignificant, but moderate positive relationship between type II fiber area and FR_MAX_ versus RT slope. Finally, there were also several strong relationships revealing the interplay between RT, FR_MEAN_, FR_MAX_, and MUAP_PP_ with type II fiber area of the vastus lateralis. Therefore, these results suggest that although the onion skin control scheme was observed in all subjects, the specific control scheme parameters (i.e., FR vs. RT slopes and/or intercepts) were correlated with muscle fiber phenotype.

While both the onion skin and reverse onion skin theories have been proposed as valid models to explain MU firing behavior, the data from the present investigation lends support to the onion skin theory (De Luca and Hostage 2010), as the larger, high‐threshold MUs in this investigation exhibited lower firing rates than the smaller, low‐threshold MUs observed during the contraction. This is evidenced by a strong, negative slope in both the FR_MEAN_ versus RT (*r* range = −0.81 to −0.95) and FR_MAX_ versus RT (*r* range = −0.81 to −0.96) relationships for all subjects. However, our data suggests that both the *y*‐intercepts and slopes of these relationships are related to an individual's fiber‐type composition, supporting the work of Herda et al. (2015) and Trevino et al. (2016). Specifically, we observed a significant negative correlation between type II fiber area and FR_MEAN_ versus RT *y*‐intercept (*R* ^2^ = 0.43), as well as a strong, direct relationship between type II fiber area and FR_MEAN_ versus RT slope (*R* ^2^ = 0.31). Similarly, we observed a significant negative correlation between type II fiber area and FR_MAX_ versus RT *y*‐intercept (*R* ^2^ = 0.71), and a strong, direct relationship between type II fiber area and FR_MAX_ versus RT slope (*R* ^2^ = 0.25).

**Figure 6:**
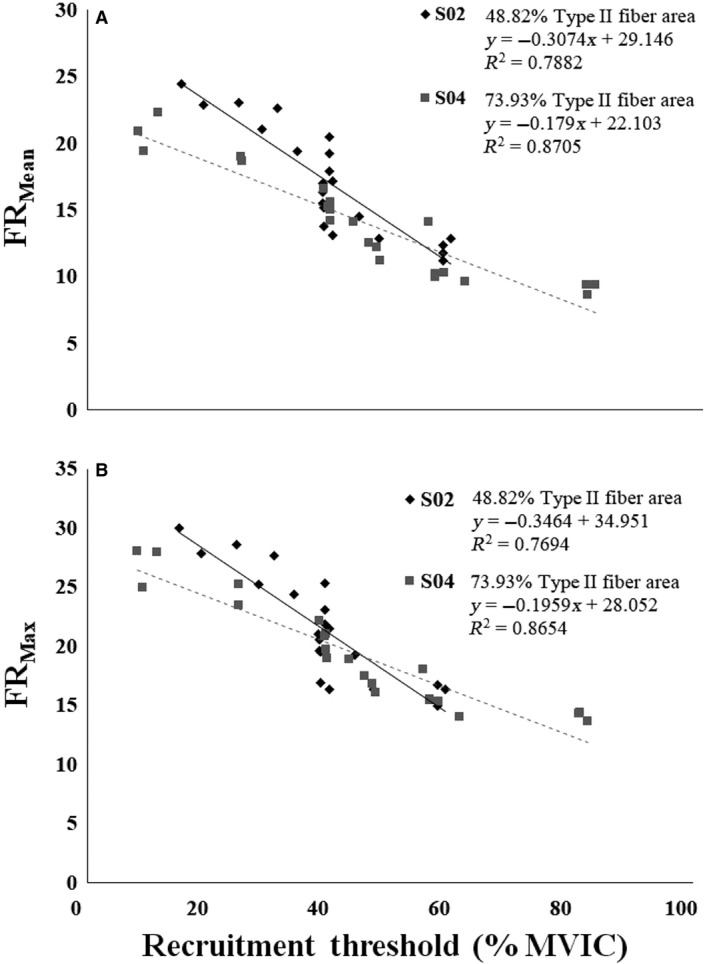
(A) Mean firing rate (FR_Mean_) versus recruitment threshold (RT) and (B) maximal firing rate (FR_Max_) versus RT relationships for two representative subjects who displayed different fiber‐type dominance. The type II fiber area for subject S02 (data represented with black diamonds and a black line) was 48.8%, whereas the type II fiber area for subject S04 (data represented with gray squares and a gray dashed line) was 73.9%.

It has been suggested that the *y*‐intercept of the FR versus RT relationship reflects the maximal sustainable firing rate of the lowest threshold MUs in the MU pool (Trevino et al. 2016). Therefore, the inverse relationship between type II MHC content and FR versus RT *y*‐intercepts suggests that the earliest recruited MUs have lower maximal sustainable firing rates in subjects with greater type II MHC content. However, the direct relationship between type II MHC content and FR versus RT slopes indicates that the higher threshold MUs have higher firing rates in individuals with greater type II MHC content. Interestingly, this may confer advantages that optimize both the maximal force generating capacity and the ability to sustain MU activity at high levels of excitation. This is illustrated in Figure 6, which displays both the FR_MEAN_ versus RT (Fig. 6A) and FR_MAX_ versus RT (Fig. 6B) relationships for representative type I and a type II MHC dominant subjects. However, our results appear to contradict the findings of previous investigations that have reported significant relationships between skeletal muscle phenotype and MU firing behavior. Although Herda et al. (2015) and Trevino et al. (2016) reported the same motor control scheme (i.e., onion skin), both authors reported more negative slopes for individuals with greater type II MHC content. These differences, however, may largely be due to the fact that both Herda et al. (2015) and Trevino et al. (2016) examined MU behavior during submaximal target forces (40% and/or 70% MVIC); whereas in the present investigation MU behavior was examined during a maximal contraction. Thus, while the results of these studies may appear contradictory, it is likely that the degree of MU pool excitation is influencing the direction of the relationships between type II MHC content and FR versus RT slopes and/or *y*‐intercepts. In support of this hypothesis, both De Luca and Hostage (2010) and Hu et al. (2013) previously demonstrated that as target force increases, the slope of the FR versus RT relationship flattens (i.e., becomes less negative). This can be clearly be seen in Figure 6 of this manuscript, as well as the data (their Figure 10) presented by Hu et al. (2013). Thus, we contend that our results are not contradictory to those of Herda et al. (2015) and Trevino et al. (2016) but are rather a function of the input excitation level.

In addition to the inverse direction of the relationships between type II MHC content and the *y*‐intercepts and/or slopes between our data and previous investigations (Herda et al. 2015; Trevino et al. 2016), the relationships observed in our study were not as strong as those in previous investigations. However, again, this is likely a function of greater excitation to the MU pool in the present investigation when compared to previous studies. For example, Hu et al. (2014a) reported a significant reduction in *R* ^2^ values and decreasing slope coefficients for FR versus RT relationships with increasing isometric contraction intensities. In addition, there is a compression of MU firing rates at higher contraction intensities as excitation to the MU pool increases. This compression results in greater homogeneity of firing rates across the RT spectrum, which would reduce the correlation coefficient of the FR versus RT relationship. Finally, as contraction intensities increase and larger MUs are recruited, there is greater superposition of individual MU action potentials in the surface EMG signal. This superposition results primarily in the inability to detect the action potentials of lower threshold MUs (Enoka and Duchateau 2015). This may also cause an increase in the homogeneity of the MUs detected during high force contractions, although we only included subjects who had a RT range of 20% MVIC or greater. Despite these factors, our data expand on the results of Herda et al. (2015) and Trevino et al. (2016) and suggest that muscle phenotype is indeed related to the motor control scheme of the muscle during maximal contractions.

Recently, Del Vecchio et al. (2017) reported that the relationship between MUAP_PP_ and RT was weak and highly variable (*R* ^2^ = 0.39 ± 0.24). In contrast, there was a significant, positive relationship between RT and MUAP_PP_ for each subject in this study (*R* ^2^ = 0.81 ± 0.08). That is, as the force at which a MU was recruited increased, a concomitant increase in its action potential size occurred for every subject. Although outside the scope of this study, there are several possible reasons for the differences observed between these studies, which include (but may not be limited to) differences in the recording technology, methodology used to analyze the signals, and the fiber‐type composition of the muscles studied. Regardless, significant RT versus MUAP_PP_ relationships (*R* ^2^ range = 0.64–0.92) were observed for all subjects independent of their fiber‐type composition. As the physical properties of the motor neuron have been shown to influence the contractile properties of the muscle fibers (Buller et al. 1960; Gundersen 1998), our data suggests that the slope and intercept of the RT versus MUAP_PP_ relationships explained 26% of the variance in skeletal muscle fiber‐type content. Specifically, our analysis suggests that those with higher type II fiber area exhibit smaller MUAPs for the lowest threshold MUs with a more dramatic increase in MUAP_PP_ across the RT spectrum than those with lower type II fiber area. Type‐II fibers have been shown to display greater cross‐sectional areas, as described by the fiber type‐fiber size paradox (Van Wessel et al. 2010). It has been suggested that bigger fibers will demonstrate MUAP_PP_ with greater amplitudes because the current generated by the action potential is dependent on the membrane area (Hakansson 1956; Pope et al. 2016). Interestingly, Pope et al. (2016) previously reported a preferential increase in MUAP_PP_ of high‐threshold MUs following resistance training, and the change in the slope of the MUAP_PP_ versus RT relationship was related to the change in whole muscle cross‐sectional area. The authors hypothesized that fiber‐type composition played a role in the degree of change. Taken together, these data may suggest that MHC content influences, to some degree, the relationship between MUAP_PP_ and RT (or vice versa).

Na^+^–K^+^ ATPase enzymes (i.e., ATPase‐*α*, ATPase‐*β*) play integral roles in numerous sensory and motor functions and may be a surrogate measure of the number of functional Na^+^/K^+^ pumps within skeletal muscle (for further detail please see review by Pirkmajer and Chibalin (2016)). Although some conflicting data exists, Na^+^/K^+^ ATPase‐*β* has been shown to be integral in the development and maturation of Na^+^/K^+^ pumps (Ackermann and Geering 1990; Geering 2008). As decreased activity of Na^+^/K^+^ pump function has been implicated in muscle fatigue during exercise (Clausen and Nielsen 2007), increased Na^+^/K^+^ pump content within the muscle may delay the accumulation of fatigue by preventing excessive loss of K^+^. This, in turn, may allow for increased exercise capacity and a maintenance of cell membrane excitability. Although speculative, this may provide a plausible explanation for the significant positive relationship between ATPase‐*β* and MVIC strength seen in this study. The *α*‐subunit, however, contains the ATP‐binding site, which is integral to the enzyme's performance of catalytic and transport activities. He et al. (2001) reported that mice expressing more of the *α*‐2 isoform produced greater force than mice expressing more of the *α*‐1 isoform. The DSHB antibody used in this study was specific to the *α*‐1 isoform, therefore the results of He et al. (2001) provide plausible support for the significant negative relationship between type II fiber area and ATPase‐*α* in this study. Furthermore, these data lend further support to the notion that skeletal muscle phenotype (i.e., physical properties of the MU pool) are strongly correlated with, and perhaps determined by, MU behavior. However, these preliminary results should be interpreted with caution until more comprehensive investigations further delineate the relationship between phenotype, enzyme activity, and MU behavior.

## Conclusions

The results of this investigation support the theory that skeletal muscle phenotype is strongly related to MU behavior in vivo. Specifically, while all subjects demonstrated an onion skin pattern of MU behavior, where MU firing rates were greatest for low‐threshold MUs and lowest for high‐threshold MUs. Interestingly, subjects with greater Type II fiber areas tended to display lower firing rates for the earliest recruited MUs, but higher firing rates for the latest recruited MUs compared to subjects who had lower Type II fiber areas. While all subjects displayed a significant relationship between action potential size and recruitment threshold, our data suggest that this relationship may also be influenced by an individual's fiber‐type composition, perhaps due to the tendency for type II fibers to display greater cross‐sectional areas. Therefore, it appears that the firing rate characteristics of the MU pool influence the physical properties of the motor unit pool (i.e., muscle phenotype) in humans. However, as correlation does not imply causation, our preliminary results should be interpreted carefully.

## Conflict of Interest

None declared.
